# Physical Exercise Ameliorates Anxiety, Depression and Sleep Quality in College Students: Experimental Evidence from Exercise Intensity and Frequency

**DOI:** 10.3390/bs12030061

**Published:** 2022-02-25

**Authors:** Chaoxin Ji, Jun Yang, Lin Lin, Song Chen

**Affiliations:** 1 Department of Physical Education, Northeastern University, Shenyang 110819, China; chensong@pe.neu.edu.cn; 2 College of Information Science and Engineering, Northeastern University, Shenyang 110819, China; yangjun@mail.neu.edu.cn; 3 School of Social and Political Science, University of Glasgow, Glasgow G12 8QQ, UK; 25140291@student.gla.ac.uk

**Keywords:** anxiety, depression, sleep quality, exercise intensity, exercise frequency

## Abstract

Background: The main purpose of this study was to investigate the effects of exercise intensity and exercise frequency on anxiety, depression and sleep quality in college students. Methods: All participants came from a university in northeastern China. All participants were tested for Diagnostic and Statistical Manual of Mental Disorders (DSM-5) and were diagnosed with anxiety disorders. The research subjects were divided into six groups, namely the low-intensity and low-frequency group (group 1), low-intensity and moderate-frequency group (group 2), low-intensity and high-frequency group (group 3), high-intensity and low-frequency group (group 4), and high-intensity and moderate-frequency group (group 5) and high-intensity and high-frequency group (group 6). The duration of each physical exercise for each group was 1 h. Participants’ exercise intensity was monitored using Polar H10 HR sensors and the Borg RPE scale. The experiment was carried out for a total of 6 weeks. The researchers conducted pre- and post-test scores on the subjects’ anxiety, depression and sleep quality through questionnaires. Results: Exercise intensity improved anxiety and decreased symptoms of depression better than exercise frequency; sleep quality was more closely related to exercise intensity. Conclusion: Exercise intensity and exercise frequency have different effects on anxiety, depression and sleep quality improvement, indicating that exercise intensity and exercise frequency have different effects on anxiety, depression and sleep quality of college students.

## 1. Introduction

Anxiety is an irritable emotion caused by excessive worry about one’s safety, fate and future. Since the COVID-19 pandemic, more and more individuals have started to show anxiety symptoms. College students are a special group. Because college students have academic pressure and employment pressure, college students may be more prone to anxiety symptoms. A study by Zhan showed that about 20.6% of college students in China have anxiety symptoms [1]. An online survey found that about 18.2% of college students in China suffer from anxiety symptoms, but most of them have mild anxiety symptoms [2]. It can be seen that the proportion of individuals with anxiety symptoms in college students is still relatively large. The occurrence of anxiety is related to many factors, but most studies believe that stress is an important factor causing anxiety, and patients with anxiety disorders are often accompanied by symptoms of insomnia. Some studies believe that anxiety and depression are highly comorbid [3,4], and patients with anxiety disorder often suffer from depression at the same time [5]. Suicidal behavior may occur if the patient suffers from severe anxiety and depression [6,7]. For the treatment of anxiety and depression, cognitive behavioral therapy (CBT) has been widely used. Although the CBT is a simple treatment with no side effects, patients may give up on treatment due to long wait times. More and more studies have shown that physical exercise can effectively relieve anxiety symptoms and depressive symptoms of patients with anxiety disorder [8]. Moreover, exercise can also improve the sleep quality of patients [9]. Physiological and psychological factors may be involved in the mechanism by which exercise relieves anxiety and depression [10]. More and more evidence now shows that in addition to aerobic exercise effectively relieving anxiety and depressive symptoms [11], other exercise methods such as application of resistance training can also effectively relieve anxiety in patients [12]. However, there are still doubts about the effectiveness of exercises in relieving anxiety and depression. Meta-analysis evidence shows that exercises have no or limited effect on improvement in depression and anxiety symptoms [13,14]. However, in contrast, a meta study suggests that a single yoga practice can effectively relieve anxiety [15]. In previous studies, the reason for the conflicting research results may be that people use different exercise interventions, such as different exercise intensity and different exercise frequency caused by the result deviation. Studies have shown that both low-intensity and moderate-intensity exercise can effectively improve sleep quality in older adults [16]. Exercise frequency may be associated with anxiety and depression, but this relationship is not clear [17]. Research has shown that exercise frequency at least once or twice a week significantly reduced anxiety and depression [18]. Therefore, we can assume that both exercise intensity and exercise frequency will have some influence on the study results. High-quality research is urgently needed to provide insight into the relationship between exercise and anxiety, depression, and sleep quality.

To explore the effects of exercises on anxiety, depression, and sleep quality, exercise intensity and exercise frequency are incorporated into this study. The study assumes that all exercise combinations can affect the anxiety, depression and sleep quality of college students, but the effect sizes are different. Among them, the high-intensity and high-frequency group has the best improvement effect, and the low-intensity and low-frequency group has the lowest improvement effect.

## 2. Materials and Methods

### 2.1. Participants

A priori power analysis (G*Power 3.1.9.7) was used to calculate the study’s required sample size. The parameters we chose were: (1) effect size f = 0.5; (2) α errprob of 0.05; (3) power of 0.85; (4) number of groups of 6; (5) number of measures of 2. After calculation, a sample size of at least 64 can yield statistical significance.

**Figure 1:**
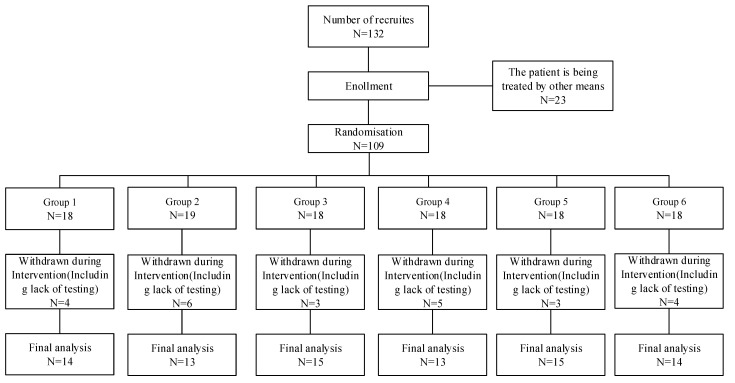
Grouping and final test of college students participating in the experiment.

This study was a controlled study to explore the effects of different exercise intensity and frequency on anxiety, depression and sleep quality of college students with anxiety disorder. College students participating in this study were recruited at the College Student Health Guidance Center. Recruitment was based on screening according to the Diagnostic and Statistical Manual of Mental Disorders (DSM-5) Anxiety Diagnostic Principles, which include separation anxiety disorder, generalized anxiety disorder, panic disorder, and social anxiety disorder. Participants were given oral and written notice at the time of recruitment and then were asked to sign a written informed consent. College students with anxiety disorders included in this study ranged in age from 19 to 29 years old, and included undergraduate, master, and doctorate students. The criteria for inclusion in this study were patients who did not use anti-anxiety medication; those with physical illnesses were excluded; those who had suffered serious physical injuries (such as fractures) within one year were excluded; those with severe mental illness diseases (psychosis) were excluded; patients who had been assessed by the Health Guidance Center as unsuitable to participate in the experiment were excluded. Additional exclusion criteria were subjects who did not complete the experiment in accordance with the experimental regulations, dropped out halfway, or exercised more than once a week outside the experiment. A total of 132 individuals were recruited at the beginning of this study. After screening, 23 individuals who did not meet the criteria of the study were excluded. Then the remaining 109 individuals were divided into 6 groups according to the random grouping software (version 1.0) (China, the LEO software studio), and sex and age were randomly distributed to ensure homogeneity. The 6 groups (low/high exercise intensity × low/moderate/high exercise frequency) were given the exercise intervention for 6 weeks (60 min each time) to observe the effects of different combinations of exercise intensity and frequency on anxiety, depression and sleep quality of college students. After the experiment, 25 individuals were excluded, and a total of 84 individuals were included in the final analysis, including 51 males and 33 females. The test samples met the needs of the study (Figure 1).

### 2.2. Study Methodology

The 6 groups were tested according to the combination of exercise intensity and frequency. The exercise intensity was divided into two grades, namely low intensity and high intensity, and the exercise frequency was divided into three grades, namely low (once a week), moderate (twice a week) and high (three times a week). According to the combination method, the 6 groups were: low-intensity and low-frequency group (group 1), low-intensity and moderate-frequency group (group 2), low-intensity and high-frequency group (group 3), high-intensity and low-frequency group (group 4), and high-intensity and moderate-frequency group (group 5) and high-intensity and high-frequency group (group 6).

All participants performed cardio training and resistance training, but the intensity and frequency varied. The exercise intensity was monitored using Polar H10 HR sensors (Polar Electro, Kempele, Finland) and the Borg RPE scale for real-time monitoring of the subjects’ exercise intensity. Low intensity was defined as Brog RPE10–13 and below 59% of maximum heart rate. High intensity was defined as Brog RPE of 14–18 and 60–95% of maximum heart rate. The training program consisted of a total of 8 repetitions of circuit training, repeated three times. Cardiorespiratory exercises included high leg running, small step running, lunges, and skipping rope. Resistance training exercises included planks, sit-ups, push-ups, and elastic band exercises. The experimental time for each group was 1 h, including 10 min of warm-up activities and 5 min of relaxation activities. The experiment was carried out for 6 weeks. During the experiment, the subjects who did not meet the experimental conditions were promptly excluded. This study was approved by the Ethics Committee of Northeastern University (EC2020B014). The study procedure was in accordance with the ethical standards of the institutional and national research committee and with the 1964 Helsinki declaration and its later amendments or comparable ethical standards.

### 2.3. Measuring Tools

The Beck Anxiety Inventory (BAI): the Beck Anxiety Inventory (BAI) was used to measure the anxiety of the subjects. The scale consisted of 21 items, and the scores of each item were nothing (0p), slightly (1p), moderately (2p) and severely (3p). Total scores: nothing/mild (0–15), moderately (16–25) and severely (26–63). Studies had shown that BAI had high reliability and validity and can be well applied to anxiety measurement in anxious individuals and normal individuals [19,20].

Self-rating depression scale (SDS): the self-rating depression scale (SDS) was used to assess the severity of perceived depression. The SDS consists of 20 items, and the scores of each item range from 1 to 4. Participants need to multiply their total score by 1.25 to be converted into the China diagnostic criteria for depression. Depressive symptoms were diagnosed if the SDS scores were higher than 53. The evaluation criteria for China’s SDS scores were as follows: the total scores ranged from (53–62) mild depression, (63–72) moderate depression and (73–100) severe depression [21]. It was reported that the reliability and validity of this scale for measuring depression in humans were high [22].

Pittsburgh sleep quality index (PSQI): the scale consists of 19 self-assessed items and 5 other-assessed items, of which the 19th self-assessed item and 5 other-assessed items were not scored. There were 7 components in the 18 items, which were sleep quality, sleep onset time, sleep time, sleep efficiency, sleep disorder, hypnotic medication, and daytime dysfunction. The total PSQI scores ranges from 0 to 21, and the higher the score, the worse the sleep quality. PSQI has high reliability and validity [23,24]. Studies had shown that PSQI can be well applied to sleep monitoring in Chinese people [25].

### 2.4. Statistical Analysis

SPSS 26.0 was used for statistical analysis of data. First, all means and standard deviations were statistically analyzed using standardized statistical methods. The normal distribution of the data was obtained using the Shapiro–Wilk test. The test was performed using a mixed ANOVA of 2 exercise intensity (low/high) × 2 time (pre/post) × 3 frequency (low/moderate/high). The test time was the within-group factor, and the exercise intensity and frequency were the between-group factors. Measured BAI and SDS were the dependent variables. The sphericity test was performed using Mauchly. If the results of the sphericity test were not met, the analysis was performed using Greenhouse–Geisser. For significant differences, Bonferroni back testing analysis was used. Partial Eta squared ($\eta_{p}^{2}$) calculated the effect sizes of significant main effects and interactions.

## 3. Results

### 3.1. Participant Characteristics

The relevant demographic information is shown in Table 1, including gender (χ^2^(5) = 1.832, *p >* 0.05), age (F(5,78) = 1.112, *p >* 0.05), body height (F(5,78) = 1.353, *p >* 0.05), body weight (F(5,78) = 1.097, *p >* 0.05), BMI (F(5,78) = 1.314, *p >* 0.05), resting heart rate (F(5,78) = 1.513, *p >* 0.05). It was shown that there was no significant difference among the six groups, indicating good consistency.

### 3.2. Baseline Analysis of Anxiety Symptoms, Depression Symptoms and Sleep Quality of College Students Pre-Test

**Figure 2:**
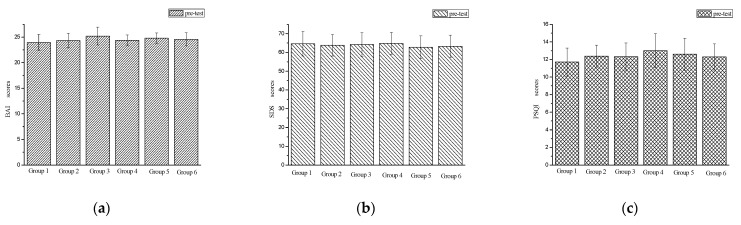
Pre-test results of participants’ anxiety, depression and sleep quality. (**a**) Pre-test results of participants’ anxiety; (**b**) pre-test results of participants’ depression; (**c**) pre-test results of participants’ sleep quality.

Before training, the BAI, SDS and PSQI scores of each group were analyzed by variance analysis, and it was found that: BAI (F(5,78) = 1.445, *p* = 0.217), SDS (F(5,78) = 1.341, *p* = 0.256) and PSQI (F(5,78) = 1.410, *p* = 0.230) pre-test scores were not significant (*p* > 0.05). This shows that there was no significant difference in the anxiety level, depression level and sleep quality level of the college students in each group before training (Figure 2). In order to explore the effect of exercise intensity and frequency on anxiety symptoms and depressive symptoms of college students, a mixed ANOVA analysis of 2 exercise intensity (low/high) × 2 time (pre/post) × 3 frequency (low/moderate/high) was used. Exercise intensity and frequency were used as between-subject variables, measurement time as the within-subject variable, and the dependent variables were BAI, SDS, and PSQI.

#### 3.2.1. Effects of Exercise Intensity and Frequency on Anxiety Symptoms of College Students

**Figure 3:**
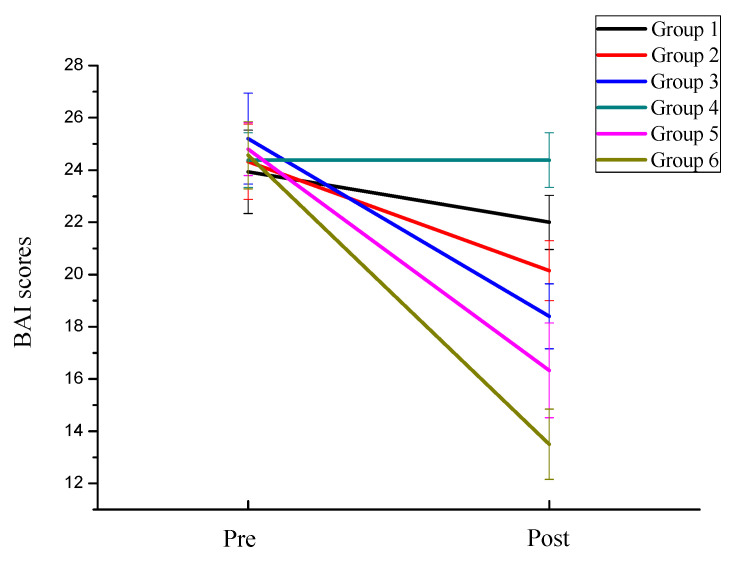
The trend of anxiety symptoms of the six groups of subjects.

In order to investigate the effects of exercise intensity and exercise frequency on the anxiety symptoms of college students, the mixed ANOVA analysis found that the main effect of anxiety measurement time was significant, F(1,78) = 1027.373, *p <* 0.001, $\eta_{p}^{2}=0.929$. Using back testing analysis, it was found that the anxiety post-test (M = 18.13, SD = 2.944) was significantly better than that pre-test (M = 24.55, SD = 1.401). The main effect of anxiety exercise intensity was significant, F(1,82) = 454.715, *p <* 0.001, $\eta_{p}^{2}=0.847$. The main effect of exercise frequency was significant, F(1,87) = 447.556, *p <* 0.001, $\eta_{p}^{2}=0.846$. Using back testing analysis, it was found that before and after training, the anxiety improvement of high exercise intensity groups was significantly higher than that of low exercise intensity groups, and the improvement of anxiety of high frequency groups was significantly higher than that of moderate frequency groups. Both were higher than those of low frequency groups (Figure 3). The interaction between measurement time and exercise intensity was significant, F(1,82) = 46.812, *p <* 0.001, $\eta_{p}^{2}=0.363$. Further simple effect analysis showed that after training, the anxiety improvement of the high exercise intensity groups (M = 16.12, SD = 2.391) was higher than that of the low exercise intensity groups (M = 20.14, SD = 1.882), F(1,82) = 73.465, *p <* 0.001, $\eta_{p}^{2}=0.473$. The interaction between measurement time and exercise frequency was significant, F(2,81) = 24.213, *p <* 0.001, $\eta_{p}^{2}=0.347$. Further simple effect analysis showed that after training, the improvement of anxiety symptoms with high frequency groups (M = 16.03, SD = 2.796) was higher than moderate frequency (M = 18.11, SD = 2.166), both higher than anxiety improvement with low frequency groups (M = 20.41, SD = 2.024), F(2,81) = 23.958, *p <* 0.001, $\eta_{p}^{2}=0.372$. Moreover, the effect of exercise intensity ($\eta_{p}^{2}=0.363$) on anxiety symptoms was higher than that of exercise frequency ($\eta_{p}^{2}=0.374$).

#### 3.2.2. Effects of Exercise Intensity and Frequency on Depression Symptoms of College Students

**Figure 4:**
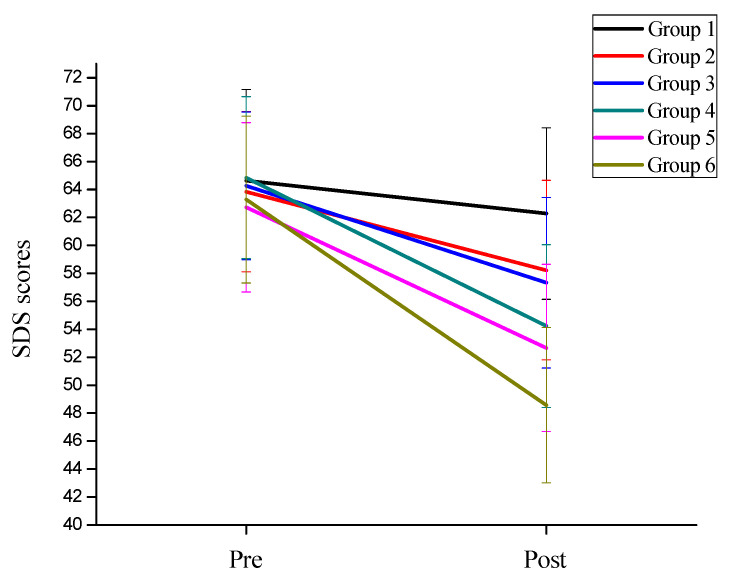
The trend of depression symptoms of the six groups of subjects.

In order to investigate the effects of exercise intensity and exercise frequency on the depression symptoms of college students, the mixed ANOVA analysis found that the main effect of measurement time on depression symptoms was significant, F(1,78) = 1240.136, *p <* 0.001, $\eta_{p}^{2}=0.941$. Using back testing analysis, it was found that the depression improvement post-test (M = 55.52, SD = 4.540) was significantly better than that measured pre-test (M = 64.27, SD = 3.219). The main effect of exercise intensity on depression symptoms was significant, F(1,82) = 779.545, *p <* 0.001, $\eta_{p}^{2}=0.905$. Using back testing analysis, it was found that the depression improvement of high-intensity groups was significantly better than that of low-intensity groups. The main effect of training frequency on depression symptoms was significant, F(1,81) = 323.876, *p <* 0.001, $\eta_{p}^{2}=0.800$. Using back testing analysis, the improvement of depression in the high-frequency groups was significantly higher than that in the moderate-frequency groups, and both were higher than those in the low-frequency groups (Figure 4). The interaction between measurement time and exercise intensity was significant, F(1,82) = 143.182, *p <* 0.001, $\eta_{p}^{2}=0.636$. After further simple effects analysis, it was found that after training, the depression improvement in the high-intensity groups (M = 51.79, SD = 2.426) was significantly higher than that in the low-intensity groups (M = 59.26, SD = 2.470). F(1,82) = 179.170, *p <* 0.001, $\eta_{p}^{2}=0.686$. The interaction between measurement time and exercise frequency was significant, F(2,81) = 6.878, *p* = 0.002, $\eta_{p}^{2}=0.415$. After further simple effect analysis, it was found that after training, the depression improvement in the high-frequency groups (M = 53.10, SD = 4.624) was significantly higher than that in the moderate-frequency groups (M = 55.25, SD = 3.063), and both were higher than those in the low-frequency groups (M = 58.41, SD = 4.218), F(2,81) = 12.216, *p <* 0.001, $\eta_{p}^{2}=0.232$. Additionally, it can be found that the effect size of depression symptom exercise intensity ($\eta_{p}^{2}=0.636$) was higher than that of exercise frequency ($\eta_{p}^{2}=0.145$).

#### 3.2.3. Effects of Exercise Intensity and Frequency on Sleep Quality Symptoms of College Students

**Figure 5:**
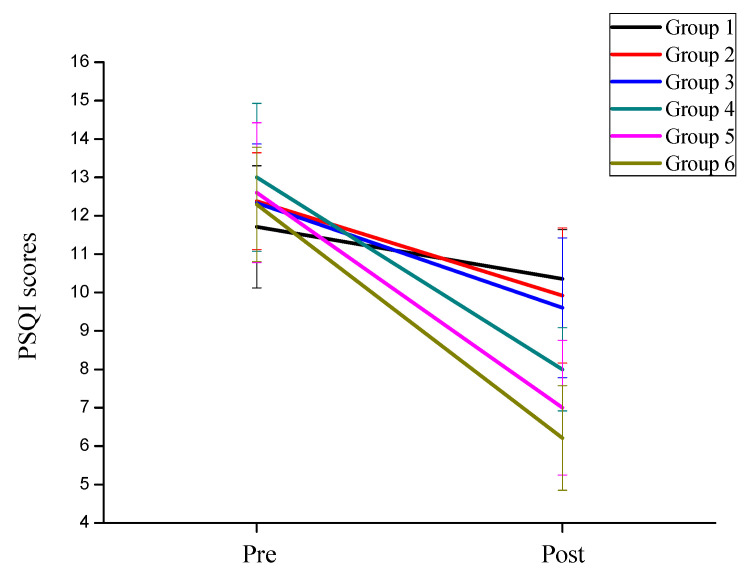
The trend of sleep quality of the six groups of subjects.

In order to investigate the effects of exercise intensity and exercise frequency on the depressive symptoms of college students, the mixed ANOVA analysis found that the main effect of measurement time on sleep quality was significant, F(1,78) = 1240.136, *p <* 0.001, $\eta_{p}^{2}=0.869$. Using back testing analysis, the sleep quality post-test (M = 8.604, SD = 0.105) was significantly higher than that pre-test (M = 12.386, SD = 0.143). The main effect of exercise intensity on sleep quality was significant, F(1,82) = 467.264, *p <* 0.001, $\eta_{p}^{2}=0.851$. Using back testing analysis, it was found that the sleep quality improvement effect of the high-intensity groups was significantly greater than that of the low-intensity groups (Figure 5). The interaction between measurement time and exercise intensity was significant, F(1,82) = 83.685, *p <* 0.001, $\eta_{p}^{2}=0.505$. Further simple effect analysis showed that after training, the improvement of sleep quality in the high-intensity groups (M = 7.21, SD = 1.353) was significantly higher than that in the low-intensity groups (M = 9.95, SD = 0.936), F(1,82) = 154.884, *p <* 0.001, $\eta_{p}^{2}=0.654$. The main effect of exercise frequency for depression was significant F(1,81) = 240.436, *p <* 0.001, $\eta_{p}^{2}=0.748$. Using back testing analysis, it was found that the sleep quality improvement effect of the high-frequency groups was significantly higher than that of the moderate-frequency groups, and both were higher than that of the low-frequency groups. The interaction between measurement time and exercise frequency was not significant F(2,81) = 2.315, *p* = 0.105, $\eta_{p}^{2}=0.054$. It showed that the frequency of exercise was not the main factor leading to the change of sleep quality.

## 4. Discussion

This study investigated the effects of exercise intensity and exercise frequency on anxiety, depression, and sleep quality in college students. Studies had shown that physical exercise can affect the anxiety, depression and sleep quality of college students, but there were differences in the extent of the effect. In the measurement of anxiety symptoms, it was found that both exercise intensity and exercise frequency will affect the anxiety symptoms of college students, but the effect of exercise intensity was higher than that of exercise frequency. Regarding the improvement effect of depressive symptoms, it was also found that the effect size of exercise intensity was higher than that of exercise frequency. In the study on the improvement of sleep quality, it was found that exercise intensity was the main factor for improving sleep quality, while exercise frequency did not lead to changes in sleep quality. Numerous studies have demonstrated beneficial effects of physical activity on anxiety [26], depression [27], and sleep quality [28]. Additional intervention using physical exercise for patients with depression can effectively relieve depressive symptoms. At present, most of the interventions are 60 min each time and three times a week [29]. However, the overall duration of the intervention in the current study was inconsistent, and the approximate intervention duration was more than 4 weeks and less than 24 weeks. Because of the factors of the duration of intervention, the results may be inconsistent for each study. This study indicated that physical exercise can improve college students’ anxiety, depression and sleep quality, and we observed the obvious dose–response effect of exercise intensity on anxiety and depression. Moreover, not only the dose–response effect of exercise intensity, but also the dose–response effect of exercise frequency was observed in this paper. However, in terms of exercise frequency, the research in this paper showed that exercise frequency can improve anxiety and decrease symptoms of depression. Moreover, the effect of exercise frequency on anxiety and depression was significantly weaker than that of exercise intensity. Our study also found that exercise frequency had no effect on sleep quality. Henriksson believes that both low-intensity and high-intensity physical exercise can improve anxiety and decrease symptoms of depression, and high-intensity exercise can improve anxiety and depression better than low-intensity exercise, but there was no significant difference [30]. The findings of our study contrast with that of Henriksson. The reason for this result may be that the groups of subjects were different. In Henriksson’s study, the ages of subjects were quite different. The responses of exercise intensity in young people and old people was different, thus causing differences. In future research, we should pay attention to the factor of age. Murphy’s study showed that college students who regularly take part in physical exercise have lower anxiety and depression symptoms [26]. Anderson’s research showed that exercise frequency was intrinsically related to anxiety, depression, and stress, and individuals with high exercise frequency have less anxiety, depression, and stress symptoms than individuals with low exercise frequency [31]. The research in this paper confirms this view. Moreover, some studies suggested that exercise frequency can lead to changes in some mental diseases. For example, Adamsde’s research showed that exercise frequency had an obvious relationship with the incidence of post-traumatic emergency disorder (PTSD), and the incidence of PTSD in individuals who do not exercise was twice that of individuals who exercise every day [32]. One of the current explanations for why physical exercise can effectively relieve people’s anxiety and depression symptoms was that exercise can promote the increase in brain-derived neurotrophic factor (BDNF). The increase in BDNF promotes neuroplasticity, neuron growth and differentiation, so it can effectively relieve anxiety and depression [33]. However, Johnston’s research pointed out that team exercise can effectively alleviate the anxiety and depression of college students, and individual physical activities have little effect on the anxiety of college students [34], which pointed out that the main reason for the influence of exercise on anxiety and depression may be team exercise. Because most of the studies were group studies and there was a lack of case studies, it is necessary to pay attention to the difference between team sports and individual sports in future studies. Through experimental research, this paper showed that exercise intensity and exercise frequency have a certain influence on anxiety and depression, and proves that exercise intensity was the main influencing factor. In the study of exercise intervention on sleep quality, Lu found that moderate-intensity exercise can improve individuals’ sleep quality to some extent [35]. Tseng showed that 12 weeks of moderate-intensity aerobic exercise can not only significantly improve the sleep quality of the elderly, but also improve the cardiac autonomic function of the elderly [36]. Studies have shown that high-intensity interval exercise was more effective than moderate-intensity training in improving sleep quality [37]. It showed that exercise intensity can significantly affect one’s sleep quality. Similarly, in the study, we also found the effect of exercise intensity on sleep quality, that is, the higher the exercise intensity, the better the improvement effect of sleep quality, and there was a significant dose–response effect. However, the Glavin’s study pointed out that exercise frequency had an impact on sleep quality, and gender factors can also affect sleep quality [38], which was contrary to the conclusion drawn in this paper that exercise frequency did not lead to improved sleep quality. The main reason for the different results from this study may be the different exercise methods used, which caused the deviation of the results. For example, Yuan’s research found that moderate-intensity aerobic exercise three times a week can significantly improve the sleep quality of the elderly [39], while Bayan-Bravo’s research showed that low-intensity exercise had no effect on improving the sleep quality of the elderly [40]. However, there was a lack of research on the comprehensive effects of exercise intensity and frequency on anxiety, depression and sleep quality, and a large number of studies were needed to prove the improvement effect of exercise intensity and exercise frequency on anxiety, depression and sleep. Although this study showed the effects of exercise intensity and exercise frequency on college students’ anxiety, depression and sleep quality, the current study also has limitations. The first is that the measurement methods used are all self-assessment methods. The self-assessment method may be influenced by subjective emotions when filling out the questionnaire, which will lead to biases in the research results. The second is that the sample we used is limited to college students, and it is unknown whether groups different from college students, such as children and the elderly, will have the same intervention results.

## 5. Conclusions

The 6 weeks exercise intervention experiment showed that exercise can improve the anxiety, sleep quality and decrease symptoms of depression of college students. Moreover, exercise intensity and exercise frequency have different effects. Among them, exercise intensity will have an impact on anxiety, depression and sleep quality, while exercise frequency will have an impact on anxiety and depression, but has a limited impact on sleep quality. The study also found that the effect of exercise intensity on anxiety and depression was higher than that of exercise frequency. In this study, we examined the effects of physical exercise on anxiety, depression, and sleep quality in college students. This study subdivided physical exercise into exercise intensity and exercise frequency and studied the effects of different combinations of exercise intensity and exercise frequency on anxiety, depression and sleep quality of college students. It provides a new research method for the study of physical exercise intervention on anxiety, depression and sleep quality. However, this paper did not discuss the effect of different weeks of exercise on anxiety, depression and sleep quality, which can be studied in future work.

## Figures and Tables

**Table 1:** Statistics of participant characteristics (M ± SD).

	Group 1	Group 2	Group 3	Group 4	Group 5	Group 6	*p*-Value
Gender (male/female)	9/5	7/6	8/7	7/6	6/9	8/6	0.872
Age (year)	23.59 ± 2.02	24.32 ± 1.98	24.11 ± 2.87	25.08 ± 2.99	23.97 ± 2.03	24.06 ± 3.05	0.314
Body height (cm)	172.13 ± 11.12	170.34 ± 10.97	171.33 ± 9.08	168.04 ± 10.95	168.32 ± 9.03	173.11 ± 11.49	0.103
Body weight (kg)	67.32 ± 8.12	69.46 ± 9.17	67.34 ± 8.97	68.21 ± 10.03	66.35 ± 9.90	70.23 ± 9.92	0.116
BMI (kg/m^2^)	22.69 ± 2.01	23.09 ± 2.96	22.95 ± 2.53	23.12 ± 2.08	23.54 ± 3.07	23.40 ± 2.60	0.317
Resting heart rate (times/min)	76.35 ± 10.22	75.54 ± 9.79	75.76 ± 11.78	78.13 ± 10.36	76.98 ± 11.45	75.90 ± 9.06	0.203

Note: M, mean; SD, standard deviation; BMI, Body Mass Index.

## Data Availability

The data presented in this study are available on request from the corresponding author. The data are not publicly available due to privacy reasons.
